# Supplementary material for: Knowledge, practice, and impact of COVID-19 on mental health among patients with chronic health conditions at selected hospitals of Sidama regional state, Ethiopia
Source: PLoS One. 2022 Jun 3;17(6):e0269171. doi: 10.1371/journal.pone.0269171 (PMC9165860; doi:10.1371/journal.pone.0269171)
Supplement: S1 File — (DOCX) [file pone.0269171.s001.docx]

# Annex I: English version questionnaire

**Hawassa University**

**College of Medicine and Health Sciences**

**School of Public Health**

***Questionnaire designed to asses Knowledge, practice and impact of COVID-19 on mental health among patients with chronic health condition at selected hospitals of sidama regional state, Ethiopia***

**1. Information sheet**

Hello! My name is _------------------------------------------------------_. I am a data collector in a survey being conducted about COVID-19 under the College of Medicine and Health Sciences of Hawassa University. The aim of this study is to measure the level of knowledge, practices and the impact of COVID-19 on mental health among patients with chronic health condition at selected hospitals of sidama regional state of Ethiopia.

The purpose of this study is to generate information about the COVID-19 among patients with chronic health condition who are living in Sidama region. The study may help stakeholders, policy makers, responsible body, and others to take actions based on the finding. The study comprises various questions like socio-demographic, knowledge and practice toward COVID-19, and mental health. You are chosen to participate in this study by chance. Interview will take not more than 30 minutes.

We assure you that there is no risk or harm in participation of this study. All information will be kept confidentially. Name of a participant will not be written or specified. Your privacy will also be protected and no one shall know your response.

This study benefits you that, you have the right to know about sign and symptoms of COVID-19 pandemic. If you are found to have a risk factor and if you are found to be suspected for COVID-19 pandemic, you will be referred for proper advice and further diagnosis and treatment. There is no incentive or payment for participating in this research.

You have full right whether or not to participate in this study. You may respond to all questions or you may not answer to the questions you don’t want to or you may quit your participation totally at any time you want. You can ask any questions which is not clear for you.

**2. Informed consent**

As to the information given ahead, participating in this study has no risk. In order to attain the objective of the study, your participation is vital. For this reason we are requesting your free will. You are selected randomly to participate in this study and your name will not be written on this form and the information you give will never be shared to others. Your genuine response to the interviews will be very important for the purpose of the study. You have a right to refuse in responding any question or the entire question at any time you want.

I have read this form or it has been read to me in the language I comprehend and understand all condition stated above.

Are you willing to participate in this study?

Yes □ No □

If “Yes" ...proceed with the interview.

If “No” ...........thank you and end.

Name of the principal investigator: Yilkal Simachew

Co-investigator: Amanuel Ejeso, Sisay Dejene/ Mohammed Ayalew

Cell phone Number - +251-941050775 E mail:- [joemakalister123@gmail.com@gmail.com](mailto:joemakalister123@gmail.com@gmail.com)

Name of interviewer___________________ Signature__________

Data of interviewer: _______________

Result of interview: 1. Complete 2. Refused 3. Partially complete 4. Respondent not available

Cheeked by supervisor: Name______________ Signature_________ Data: _____________

**SECTION 1. SOCIODEMOGRAPHIC CHARACTERISTICS**

| **S. No** | **Questions** | **Responses** |
| --- | --- | --- |
| 001 | Sex | 1. Male 2. Female |
| 002 | Age | _____________ years |
| 003 | Educational status | 1. Unable to read & write 2. Read & write 3. Elementary 4. Second & above |
| 004 | Marital status | 1. Single 2. Married 3. Divorced 4. Widowed |
| 005 | Residence | 1. Urban 2. Rural |
| 006 | Monthly Income | _________________ETB |
| 007 | Occupations | 1. Merchant 2. Governmental employee 3. Private employee 4. Farmer 5. Housewife 6. Other ____________________________ |
| 008 | Type of chronic disease | 1. Diabetes mellitus 2. Hypertension 3. Heart disease 4. Chronic lung disease 5. Other _______________ |
| 009 | Name of Hospital | ______________________ |

**SECTION 2. QUESTIONS TO MEASURE KNOWLEDGE OF CHRONIC DISEASE PATIENTS ON COVID-19**

| S. No. | Knowledge Questions | Yes | No | I don’t know |
| --- | --- | --- | --- | --- |
| 1 | Main clinical symptoms of COVID-19 are fever, cough, shortness of breath, and fatigue |  |  |  |
| 2 | Unlike the common cold, stuffy nose, runny nose, and sneezing are less common in persons infected with the COVID-19 virus |  |  |  |
| 3 | COVID-19 symptoms appear within 2–14 days |  |  |  |
| 4 | Currently, there is no effective treatment or vaccine for COVID-2019, but early symptomatic and supportive treatment can help most patients to recover from the infection |  |  |  |
| 5 | Not all persons with COVID-19 will develop severe cases. Those who are elderly, have chronic illnesses, and with suppressed immunity are more likely to be severe cases |  |  |  |
| 6 | Touching or shaking hands of an infected person would result in the infection by the COVID-19 virus |  |  |  |
| 7 | Touching an object or surface with the virus on it, then touching your mouth, nose, or eyes with the unwashed hand would result in the infection by the COVID-19 virus |  |  |  |
| 8 | The COVID-19 virus spreads via respiratory droplets of infected individuals through the air during sneezing or coughing of infected patients |  |  |  |
| 9 | Persons with COVID-19 cannot infect the virus to others if he has no any symptom of COVID-19 |  |  |  |
| 10 | Wearing masks when moving out of home is important to prevent the infection with COVID-19 virus |  |  |  |
| 11 | Children and young adults do not need to take measures to prevent the infection by the COVID-19 virus |  |  |  |
| 12 | To prevent the COVID-19 infection, individuals should avoid going to crowded places such as public transportations, religious places, Hospitals and Workplaces |  |  |  |
| 13 | Washing hands frequently with soap and water for at least 20 seconds or use an alcohol based hand sanitizer (60%) is important to prevent infection with COVD-19 |  |  |  |
| 14 | Traveling to an infectious area or having contact with someone traveled to an area where the infection present is a risk for developing an infection |  |  |  |
| 15 | Isolation and treatment of people who are infected with the COVID-19 virus are effective ways to reduce the spread of the virus |  |  |  |
| 16 | People who have contact with someone infected with the COVID-19 virus should be immediately isolated in a proper place |  |  |  |

**SECTION 3. QUESTIONS TO MEASURE THE PRACTICE LEVEL OF COVID-19 PREVENTION**

| **S. No** | **Practice Questions** | **Yes** | **No** |
| --- | --- | --- | --- |
| 1 | Do you participate in meetings, religious activities, events, and other social gatherings or any crowded place in areas with ongoing community transmission? |  |  |
| 2 | In recent days, have you worn a mask when leaving home? |  |  |
| 3 | If yes, do you touch the front of the mask when taking it off? |  |  |
| 4 | Do you reuse a mask? |  |  |
| 5 | Do you wash your hands with soap and water frequently for at least 20seconds or use sanitizer/60% alcohol |  |  |
| 6 | Do you touch your eyes, nose, and mouth frequently with unwashed hands? |  |  |
| 7 | Do you clean and disinfect frequently touched objects and surfaces |  |  |
| 8 | Do you practice “physical distancing” by remaining 6 feet or 2 meters away from others at all times? |  |  |
| 9 | Do you use other workers’ phones, desks, offices, or other work tools and equipment? |  |  |
| 10 | Do you limit contact (such as handshakes) |  |  |
| 11 | Do you eat or drink in bars and restaurants? |  |  |
| 12 | Do you cover your nose and mouth during coughing or sneezing with the elbow or a tissue, then throw the tissue in the trash |  |  |
| 13 | Do you prefer to stay at home, in a room with the window open during the transmission period |  |  |
| 14 | Do you stay home when you were sick due to common cold-like infection during the transmission period |  |  |
| 15 | Do you listen and follow the direction of your state and local authorities? |  |  |

**SECTION 4. HOSPITAL ANXIETY AND DEPRESSION SCALE (HADS)**

| D | A |  | D | A |  |
| --- | --- | --- | --- | --- | --- |
|  |  | **I feel tense or 'wound up':** |  |  | **I feel as if I am slowed down:** |
|  | 3 | Most of the time | 3 |  | Nearly all the time |
|  | 2 | A lot of the time | 2 |  | Very often |
|  | 1 | From time to time, occasionally | 1 |  | Sometimes |
|  | 0 | Not at all | 0 |  | Not at all |
|  |  | **I still enjoy the things I used to enjoy:** |  |  | **I get a sort of frightened feeling like 'butterflies' in the stomach:** |
| 0 |  | Definitely as much |  | 0 | Not at all |
| 1 |  | Not quite so much |  | 1 | Occasionally |
| 2 |  | Only a little |  | 2 | Quit often |
| 3 |  | Hardly at all |  | 3 | Very often |
|  |  | **I get a sort of frightened feeling as if something awful is about to happen:** |  |  | **I have lost interest in my appearance:** |
|  | 3 | Very definitely and quite badly | 3 |  | Definitely |
|  | 2 | Yes, but not too badly | 2 |  | I don't take as much care as I should |
|  | 1 | A little, but it doesn't worry me | 1 |  | I may not take quite as much care |
|  | 0 | Not at all | 0 |  | I take just as much care as ever |
|  |  | **I can laugh and see the funny side of things:** |  |  | **I feel restless as I have to be on the move:** |
| 0 |  | As much as I always could |  | 3 | Very much indeed |
| 1 |  | Not quite so much now |  | 2 | Quite a lot |
| 2 |  | Definitely not so much now |  | 1 | Not very much |
| 3 |  | Not at all |  | 0 | Not at all |
|  |  | **Worrying thoughts go through my mind:** |  |  | **I look forward with enjoyment to things:** |
|  | 3 | A great deal of the time | 0 |  | As much as I ever did |
|  | 2 | A lot of the time | 1 |  | Rather less than I used to |
|  | 1 | From time to time, but not too often | 2 |  | Definitely less than I used to |
|  | 0 | Only occasionally | 3 |  | Hardly at all |
|  |  | **I feel cheerful:** |  |  | **I get sudden feelings of panic:** |
| 3 |  | Not at all |  | 3 | Very often indeed |
| 2 |  | Not often |  | 2 | Quite often |
| 1 |  | Sometimes |  | 0 | 1 Not very often |
| 0 |  | Most of the time all |  | 0 | Not at |
|  |  | **I can sit at ease and feel relaxed:** |  |  | **I can enjoy a good book or radio or TV program:** |
|  |  | Definitely |  |  | Often |
|  |  | Usually |  |  | Sometimes |
|  |  | Not often |  |  | Not often |
|  |  | Not at all |  |  | Very seldom |

Scoring: Total score: Depression (D) ___________ Anxiety (A) ______________ 0-7 = Normal 8-10 = Borderline abnormal (borderline case) 11-21 = Abnormal (case)
