# Supplementary material for: Knowledge, practice, and impact of COVID-19 on mental health among patients with chronic health conditions at selected hospitals of Sidama regional state, Ethiopia
Source: PLoS One. 2022 Jun 3;17(6):e0269171. doi: 10.1371/journal.pone.0269171 (PMC9165860; doi:10.1371/journal.pone.0269171)
Supplement: S3 File — (DOCX) [file pone.0269171.s003.docx]

Yilkal Simachew

Lecturer and Researcher

Hawassa University, Ethiopia

[joemakalister123@gmail.com](mailto:joemakalister123@gmail.com)

Editorial Department

*Journal of PLOS ONE*

[em@editorialmanager.com](mailto:em@editorialmanager.com)	May 19, 2022

**Subject:** Modification and resubmission of manuscript (PONE-D-21-17635R2)

Dear Editor

Thank you for giving us the opportunity to modify and resubmit the manuscript “Knowledge, practice and impact of COVID-19 on mental health among patients with chronic health conditions at selected hospitals of Sidama regional state, Ethiopia”, for publication in the *journal of PLOS ONE.* We would like to thank the editors for their time and facilitating the process.

We have carefully considered all suggestions and did our best to address every one of them. The changes are marked in the revised manuscript with locked track change. We have included the Editor Comments immediately after this letter and responded to them individually.

We thank you for your continued interest in our research.

Sincerely,

Yilkal Simachew

Lecturer and Researcher at Hawassa University, in Ethiopia

**JOURNAL REQUIREMENTS:

Comment 1. Please ensure that the author list and affiliations are correct on the title page of your manuscript, and that your author contributions, competing interests, and financial disclosure are correct as listed below. All of these sections will be indexed in PubMed and published by PLOS ONE as you have written them. Please email** [**plosone@plos.org**](mailto:plosone@plos.org) **if any changes to this content need to be made.**

**Response:** On the title page, the author list and affiliations are proper. The financial disclosure and competing interests are accurately mentioned. In terms of author contribution, we have only mentioned the author's participation utilizing seven roles. PLOS ONE, on the other hand, uses the CRediT Taxonomy to acknowledge the author's contribution. Now we have revised according to the PLOS ONE requirements using CRediT Taxonomy (Line # 468-479).

**Comment 2: To prevent production delays, we recommend using the Author Formatting Checklist to confirm that your paper meets PLOS ONE's typesetting requirements for References, Tables, and Figures:** [**http://journals.plos.org/plosone/s/file?id=c819/plos-one-author-formatting-checklist.docx**](http://journals.plos.org/plosone/s/file?id=c819/plos-one-author-formatting-checklist.docx).

**Response:** Based on your advice, we used the author formatting checklist to ensure that our paper met the PLOS ONE typesetting requirements for references, tables, and figures. According to the checklist results, the references and figure meet the PLOS ONE requirement, but not the table. We have checked the journal's table preparation guidelines. One of the table formatting rules is to use consistent alignment (center or left) to justify the text within the cell. Furthermore, the guideline prohibits using space to indent or justify content. Based on this, we removed the space we used to indent and justify. At the same time, we have aligned the text within the table cell to the left (line # 262-264, 277-279, 303-305, 327-329, and 351-353). Following the correction, we have used author formatting checklist and we have confirmed that our paper meet the PLOS ONE typesetting requirements for references, table and figures.

**Comment 3: To ensure your figures meet our technical requirements, please run each figure included in your submission files through the PACE tool:** [**https://pacev2.apexcovantage.com/**](https://pacev2.apexcovantage.com/)**. PACE will assess whether your figures meet our technical requirements and will fix the figure(s) or identify any problem(s) that cannot be automatically fixed. It can also convert figures to TIFF format, resize, and rename figures to meet our naming conventions.To use PACE, first register as a user. Follow the instructions on the site for assessing and converting your figure files. If you experience any difficulty using this tool or have questions about any of the figures and/or images in your paper, please inform the journal office in your response letter.**

**Response:** Thank you for your suggestion. Based on the suggestion, we have registered with PACE and uploaded the figure to see if it met the PLOS ONE requirements. According to PACE report, the figure meets the technical requirement of PLOS ONE journal; the resolution, dimension, file format, and file size are in line with the journal requirement.
