# Supplementary material for: Knowledge, practice, and impact of COVID-19 on mental health among patients with chronic health conditions at selected hospitals of Sidama regional state, Ethiopia
Source: PLoS One. 2022 Jun 3;17(6):e0269171. doi: 10.1371/journal.pone.0269171 (PMC9165860; doi:10.1371/journal.pone.0269171)
Supplement: Attachment — Submitted filename: Comments.docx [file pone.0269171.s004.docx]

REVIEWER EVALUATION.

Knowledge, practice and impact of COVID-19 on mental well health of chronic disease patients at selected hospitals in Sidama regional states in Ethiopia were evaluated. This is an interesting paper with great potential that needs minor revisions. The paper also needs through editing because at times the language used is rather unclear.

Detailed Comments.

**Abstract.**

1. In the conclusion section, author reported prevalence of good knowledge and practice to be low yet in the result section author reported more than half of the participants to have good knowledge. I suggest to be consistent in reporting the results and conclusion.

**Introduction:**

1. This section is excessively long. The authors should shorten this section.
2. Statement on lines 71 – 72 needs to be cited.
3. First statement on paragraph 2 (lines 76-77) can be integrated in first paragraph.
4. Statement on lines 80 – 81 needs to be referenced.
5. Paragraph on lines 86 – 99 needs to be focused on people with chronic medical conditions but not the older adults since the study is on people with chronic medications.

**Methods:**

1. How did you determine that one had a chronic medical condition? Was it self-reported or tests were done. This needs to be elucidated.
2. Did the study have any exclusion criteria? If yes, this needs to be stated.
3. Kindly Elaborate more on the profession of the translators. What qualification do they need to ensure accurate?
4. Should all preventative measures be given the same weight in scoring?
5. Why choose dichotomous categories for knowledge and practice as opposed to multiple categories? If choosing a dichotomous approach, why use 80% as the cut-off? Please provide justification for your approach in both cases.

**Results:**

1. Correct the table numbers in the descriptions to align with their titles.

**Discussion:**

1. Vaccines and treatments: I think you should be clearer here - i.e. There are vaccines currently approved and enrolled. There are treatments that seem to help symptoms and reduce death rates, bit there is no cure.
2. The authors should describe in detail all the biases present in the study and these appear before the conclusions.
