# Supplementary material for: Knowledge, practice, and impact of COVID-19 on mental health among patients with chronic health conditions at selected hospitals of Sidama regional state, Ethiopia
Source: PLoS One. 2022 Jun 3;17(6):e0269171. doi: 10.1371/journal.pone.0269171 (PMC9165860; doi:10.1371/journal.pone.0269171)
Supplement: Attachment — Submitted filename: Response to Reviewers.docx [file pone.0269171.s006.docx]

Yilkal Simachew

Lecturer and Researcher

Hawassa University, Ethiopia

[joemakalister123@gmail.com](mailto:joemakalister123@gmail.com)

Dr. Chong Chen

Editorial Department

*Journal of PLOS ONE*

[em@editorialmanager.com](mailto:em@editorialmanager.com)	May 14, 2022

**Subject:** Revision and resubmission of manuscript (PONE-D-21-17635R1)

Dear Dr. Chong

Thank you for giving us the opportunity to revise and resubmit the manuscript “Knowledge, practice and impact of COVID-19 on mental health among patients with chronic health conditions at selected hospitals of Sidama regional state, Ethiopia”, for publication in the *journal of PLOS ONE.* We wish to express our appreciation to the editor and the anonymous reviewers for your in-depth comments, suggestion, and corrections, which have greatly improved the manuscript. We have carefully considered all suggestions and concerns and tried our best to address every one of them. The changes are marked in the revised manuscript.

We have included the Reviewers and Editor Comments immediately after this letter and responded to them individually, indicating exactly how we addressed each concern and describing the changes we have made. Please note that reviewers’ comments are shown in **bold** type and our responses in plain type. Changes to the manuscript that have been made are marked in blue color in the revised version. The removal of text at certain locations is highlighted with ~~red~~ color in the revised manuscript.

We thank you for your continued interest in our research.

Sincerely,

Yilkal Simachew

Lecturer and Researcher at Hawassa University, in Ethiopia

**REPLY TO ACADEMIC EDITOR’S COMMENTS**

**Comment 1: Thank you for submitting your revised manuscript to PLOS ONE. The three reviewers have accepted all your responses and revisions. The manuscript, however, will benefit from English editing by either a native speaker, someone proficient in English, or a professional editing service.**

Response: Thank you for considering our revised paper for further revision, we would like to thank all reviewers for their time and valuable comments to improve our paper. We thank you for advising us to make edition to the manuscript's English writing. A native speaker edited the manuscript’s language. Based on the report of edition, we have made changes throughout the manuscript. The changes that have made found in revised manuscript with track changes.

**Comment 2: A marked-up copy of your manuscript that highlights changes made to the original version. You should upload this as a separate file labeled 'Revised Manuscript with Track Changes'.**

Response: We have uploaded a marked-up copy named “Revised Manuscript with Track Changes”.

**Comment 3: An unmarked version of your revised paper without tracked changes. You should upload this as a separate file labeled 'Manuscript'.**

Response: We have uploaded revised paper named “Manuscript”.

**Comment 4: If you would like to make changes to your financial disclosure, please include your updated statement in your cover letter.**

Response: We have not made any changes to financial disclosure.

**Comment 5: Guidelines for resubmitting your figure files are available below the reviewer comments at the end of this letter.**

Response: Thank you for your suggestion. Based on the suggestion, we have registered with PACE and uploaded the figure to see if it met the PLOS ONE requirements. Following the PACE report, we corrected the resolution, dimension, file format, and file size of figure. Now, the revised figure does fit with PLOS. We downloaded the figure from the PACE and uploaded as Fig 1, in TIFF format.

**Comment 6: If applicable, we recommend that you deposit your laboratory protocols in** [**protocols.io**](http://protocols.io/) **to enhance the reproducibility of your results. Protocols.io assigns your protocol its own identifier (DOI) so that it can be cited independently in the future. For instructions see:** [**https://journals.plos.org/plosone/s/submission-guidelines#loc-laboratory-protocols**](https://journals.plos.org/plosone/s/submission-guidelines#loc-laboratory-protocols)**.**

Response: Our study does not have any laboratory protocol to deposit.

**Comment 7: Please review your reference list to ensure that it is complete and correct. If you have cited papers that have been retracted, please include the rationale for doing so in the manuscript text, or remove these references and replace them with relevant current references. Any changes to the reference list should be mentioned in the rebuttal letter that accompanies your revised manuscript. If you need to cite a retracted article, indicate the article’s retracted status in the References list and also include a citation and full reference for the retraction notice**

**Response:** We've updated the reference list to make it complete and accurate. We updated the references to conform to the International Committee of Medical Journal Editors' reference format (ICMJE). The following is a list of updated references to ensure that it is complete and accurate, as required by the journal.

Reference number 1: According to ICMJE citation style, it is an online paper with a DOI. As a result, we have included the entire DOI next to the volume (issue) and page numbers in the revised manuscript (Revised manuscript, line #500-502).

Reference number 2: The responsible body was not mentioned in the previous format; this has been corrected in the revised version. According to the ICMJE, because the journal article is available in two languages (English and Chinese), the journal title should be presented in its original language (Chinese). We have made the necessary changes and added the DOI and PMID (Revised manuscript, line #505-509).

Reference number 3: The source is a website, but the accessed date is missing in the previous format. We have made the necessary changes (Revised manuscript, line #511-513).

Reference number 4: The source is a website, but the accessed date is missing in the previous format. We have made the necessary changes (Revised manuscript, line #516-519).

Reference number 5: The order of the authors' names was incorrect in the previous format. It also lacks the journal's name. The authors' names have been rearranged, and the journal title has been abbreviated in accordance with the NLM catalogue of NCBI journals (Revised manuscript, line #522-525).

Reference number 6: The accessed date is missing in the previous format. We have now mentioned the accessed date of the website (Revised manuscript, line #531-534).

Reference number 7: According to the ICMJE citation style, only the first word and proper nouns are capitalized in the title of the article. We have made the necessary changes. Furthermore, the journal title has been abbreviated in accordance with the NLM catalogue of NCBI journals. To make the citation complete, we've added DOI and PMID after the page number (Revised manuscript, line #538-542).

Reference number 8: In the previous format, authors are listed by their first name rather than their surname. We have now revised the author's name to include the surname first, followed by the first initial. Furthermore, the previous format lacked the journal title, which we have corrected (Revised manuscript, line #545-548).

Reference number 9: In the previous format, the journal title was written in its full extent (International Journal of Environmental Research and Public Health). We have now revised it and included the journal title in its abbreviated form as per the NLM catalogue of NCBI journals (Int J Environ Res Public Health). In addition, the DOI and PMID have been updated (Revised manuscript, line #552-555).

Reference number 11: The accessed date is missing in the previous format. We have now mentioned the website's access date (Revised manuscript, line #566-569).

Reference number 12: The author's name was not written correctly in the previous format. Furthermore, the web address was incomplete. We have now corrected the author's name and written the complete web address (Revised manuscript, line #571-572).

Reference number 14: The accessed date is missing in the previous format. We have now mentioned the website's access date (Revised manuscript, line #576-578).

Reference number 15: The arrangement of the author's name was incorrect in the previous format. We have now rearranged the author's name. Furthermore, the DOI and PMID are inserted next to the page number (Revised manuscript, line #581-584).

Page number 16: We have included the article's DOI and PMID (Revised manuscript, line #587-590).

Reference number 17: We listed all ten authors in the previous version. However, the ICMJE citation style limits the number of authors to six, followed by "et al." We made the necessary changes and added the DOI and PMID of article (Revised manuscript, line #594-597).

Reference number 18: We limited the number of authors to six and used et al (Revised manuscript, line #601-605).

Reference number 19: The previous format did not indicate that this reference was preprint. We have now added [Preprint] next to the article title. In addition, we mentioned the citation date (Revised manuscript, line #608-612).

Reference number 20: We limited the number of authors to six and used et al. In addition, we have included the article's DOI and PMID (Revised manuscript, line #616-620).

Reference number 21: We limited the number of authors to six and used et al. In addition, we have included the article's DOI and PMID (Revised manuscript, line #624-628).

Reference number 22: The previous format did not indicate that this reference was preprint. We have now added [Preprint] next to the article title (Revised manuscript, line #631-635).

Reference number 23: We have included the article's DOI and PMID (Revised manuscript, line #640-643).

Reference number 24: The list of authors' names has been updated. DOI and PMID have also been added (Revised manuscript, line #646-649).

Reference number 25: The journal title was misspelt in the previous format (Front Public Heal). We have made the necessary changes (Front Public Health) (Revised manuscript, line #652-656).

Reference number 26: The list of authors' names has been updated. DOI and PMID have also been added (Revised manuscript, line #659-662).

Reference number 27: We have included the article's DOI and PMID (Revised manuscript, line #665-668).

Reference number 28: The list of authors' names has been updated. The journal title was misspelt in the previous format. We have made the necessary changes. In addition, a DOI has been added (Revised manuscript, line #671-674).

Reference number 29: We have included the article's DOI and PMID (Revised manuscript, line #678-681).

Reference number 30: We have included the article's DOI and PMID (Revised manuscript, line #684-687).

**Response to Reviewers' comments:**

**Reviewer #2**

**Comment 1: Generally, the authors appropriated response to my comments and suggestions. However, some change is needed before publication: I believe that your manuscript could benefit by English language professional service.**

Response: Thanks for your kind comments. We appreciate your suggestion to revise the manuscript's English writing; we have addressed all concerns in this revision. The language of the manuscript was edited by a native speaker. We made changes throughout the manuscript based on the edition report. The changes found in the revised manuscript with track changes.

**Reviewer #3**

**Comment 1: Well done. All the issues raised have been sufficiently addressed. I believe the manuscript has improved greatly now.**

Response: Thank you very much.
